# Medical students’ communication skills in peer role-plays: An exploratory observational study

**DOI:** 10.4102/phcfm.v17i1.4926

**Published:** 2025-10-24

**Authors:** Jennifer Watermeyer, Johanna Beukes, Aviva Ruch, Deidré Pretorius

**Affiliations:** 1 Health Communication Research Unit, School of Human and Community Development, Faculty of Humanities, University of the Witwatersrand, Johannesburg, South Africa; 2 Unit for Undergraduate Medical Education, Faculty of Health Sciences, University of the Witwatersrand, Johannesburg, South Africa; 3 Division of Family Medicine, School of Clinical Medicine, Faculty of Health Sciences, University of the Witwatersrand, Johannesburg, South Africa

**Keywords:** communication, counselling, empathy, motivational interviewing, observational studies, South Africa, students, medical

## Abstract

**Background:**

Medical students are commonly taught two counselling protocols: breaking bad news and brief motivational interviewing for behaviour change. They must demonstrate advanced skills such as empathy, active listening, clear communication, offering support and creating a safe space for patients and their families to express their emotions. Medical students are taught communication skills through various methods, including peer role-play.

**Aim:**

This study aimed to document medical students’ communication skills as evident across recorded peer role-play scenarios and observe how students engage with this approach to practice communication skills.

**Setting:**

Final-year medical students at a medical school in Gauteng, South Africa.

**Methods:**

The study involved an observational approach to analyse 45 video- and audio-recorded student-led peer role-play scenarios that included breaking bad news and brief motivational interviewing skills, as part of an exploratory qualitative design. Thematic analysis was conducted.

**Results:**

The three main challenges students experienced were basic information giving and clinical correctness, doctor-centred versus patient-centred talk and providing psychosocial support and showing empathy. The authenticity of the peer-role-play was also a challenge.

**Conclusion:**

Making the transition from communication theory to practice may be difficult for students to achieve and learning how to integrate these complex communication skills is not straightforward. Training in communication and counselling skills must start early for medical students.

**Contribution:**

Family Medicine often takes responsibility for training communication and counselling skills in medicine, and our study can contribute to the discussion on training communication skills.

## Introduction

Two common counselling protocols that medical students are taught include breaking bad news (BBN) and brief motivational interviewing (BMI) for behaviour change. Breaking bad news is a challenging and sensitive task for healthcare providers.^1^ There are several essential skills involved in this task. These include demonstrating empathy, active listening, clear communication, offering support and creating a safe space for patients and their families to express their emotions. These skills help establish trust, ensure accurate interpretation of diagnosis and treatment and reduce misunderstandings.^2,3,4^ Brief motivational interviewing is a collaborative, patient-centred approach to behaviour change, focusing on increasing patients’ motivation. It involves engaging patients, discussing advantages and disadvantages, choosing necessary changes, considering their motivations and agreeing on a plan. Skills like reflective listening and affirmation are employed to conduct BMI.^5^

When training medical students in these skills, peer role-play (PRP) is often used since it is a cost-effective and well-received method for improving communication skills among medical students.^6^ It is less expensive than other simulation-based approaches, such as standardised patients who require remuneration.^7,8^ This form of simulation encourages empathic responses to patients, particularly when the student plays the patient role.^9^ The PRP approach has benefits because it can provide ‘a safe environment where mistakes [*are*] allowed, corrected and proper skills reiterated’^10^ (p. 75), promote ‘a 360° understanding of the doctor-patient relationship’^11^ (p. 38) and offer the opportunity for greater consideration of patient concerns.^12,13^ Peer role-play has also been criticised for its lack of authenticity, where students may struggle to fully embody the role of a patient or clinician.^14^ Students may also feel anxiety related to performing in front of peers, which can inhibit their ability to engage fully with this type of learning experience.^15^ There is also limited evidence suggesting that this approach leads to improved clinical outcomes.^8^

Studies observing students’ communication skills in PRP scenarios have often employed coding or checklist approaches to document observed communication behaviours.^9,12,16,17,18,19,20,21^ While these approaches are useful to some extent, checklists and rubrics do not reflect the interactional complexity of communication or offer a holistic, nuanced view of students’ communication skills.^22^

Our study, therefore, involved an observational sociolinguistic-based qualitative analysis of a dataset of recorded peer-role-play scenarios, utilising an interdisciplinary approach with multiple perspectives.^23^ The aims of this study were: (1) to document medical students’ communication skills as evident across the recorded peer-role-play scenarios, (2) to explore whether and how students apply the communication skills for BBN and BMI taught in tutorials and (3) to observe how students engage with PRP as a tool for practising communication skills.

## Research methods and design

### Study design

The study involved an observational approach to analyse video and audio-recorded student-led PRP scenarios as part of a previously described exploratory qualitative design,^24^ focusing especially on the behaviours of the students in the role of the doctor. These scenarios formed part of a formative assessment process.

The research team comprised members with different disciplinary expertise and a range of so-called insider and outsider perspectives.^25^ Author 1 (J.W.) is a clinical educator in speech pathology and audiology with expertise in health communication and interactional research. Author 2 (J.B.) is a research psychologist with experience in neuropsychological clinical work and health communication research. Author 3 (A.R.) is a clinician and clinical educator specialising in health education. Author 4 (D.P.) is a social scientist with expertise in psychosocial intervention, communication and counselling in health. Authors 3 (A.R.) and 4 (D.P.) were involved with the teaching and assessment of communication and clinical skills to the students involved in this project.

### Setting

Medical students at the University of the Witwatersrand are unique in that they may enter the programme as school leavers and complete a 6-year programme or enter with an undergraduate degree in their third year of study as part of the graduate entry medical programme. They learn clinical skills from the third year, including physical examination, history taking and communication; these skills are taught primarily via simulation activities. Communication skills are included in every clinical skills session (approximately 15) over the third and fourth years of study, including specific sessions that involve skills like history taking.

In the sixth year, students are taught BBN skills using the ABCDE approach.^26^ This approach involves the following: Advance preparation, Build a therapeutic environment/relationship, Communicate well, Deal with patient and family reactions, and Encourage and validate emotions. Students are taught behaviour change skills using Miller and Rollnick’s principles.^5^ They receive lectures and tutorials on BBN and BMI, wherein a scenario is role-played and students critique it. The students have opportunities to practice these skills both with their lecturers and with facilitators on their clinical learning experiences in the district circuit.

Thereafter, they are given two PRP formative assignments (the focus of this study) to practice and demonstrate their competency in these two communication skills and enable clinical educators to provide the students with formative feedback on their communication skills. All the students were briefed on expectations and the types of scenarios that would be most appropriate. The students were encouraged to use real-life examples of scenarios they had seen in their clinical encounters and then record a PRP with one student playing a doctor and another a patient. They were given the option to submit in audio or video format on both components, depending on technological ability and availability. All of the processes described here were part of the general educational programme.

Formative feedback was then provided individually to the students on their recorded PRP scenarios, using a rubric loosely based on the Calgary-Cambridge guides.^22^ It assessed verbal and non-verbal communication, questioning techniques, use of medical jargon, shared decision-making skills, clinical reasoning, active listening skills, responses to patient cues and dealing with confrontation and resistance.

Although guidelines for teaching BBN^27^ and BMI^28^ are available in the literature, limited time within the curriculum often means these skills are not prioritised. As a result, they are typically taught in a condensed manner, leaving students to further develop and refine them ‘on the job’ after graduation. The teaching approach at our university is admittedly introductory and offers merely one approach to provide students with a starting point, so they start their internship with some communication skills for BBN and BMI.

In addition, the students in this study received most of their second-year training online because of the coronavirus disease 2019 (COVID-19) pandemic, with a hybrid approach in their third year. Consequently, the pandemic significantly disrupted their opportunities for practice and clinical interaction with patients, and they entered their final year facing complex clinical skills with limited hands-on experience during the formative stages of their training. The students were, however, provided with additional practice sessions at their request.

### Study population and sampling strategy

The entire group of 339 final-year medical students was invited to provide consent for analysis of their recorded PRP scenarios, using a convenience sampling approach.

### Data collection

The scenarios were recorded during 2022. The data set (*N* = 45) comprised 21 BBN scenarios (14 video-recorded and seven audio-recorded) and 24 BMI scenarios (14 video-recorded and 10 audio-recorded) conducted by 24 male and female students, aged 23 years and older. There were no withdrawals from the study. The scenarios were an average of 6 min 55 s long, with the shortest at 3 min 39 s and the longest at 15 min 26 s. Students were instructed to aim for 5 min – 10 min per scenario.

### Data analysis

A hybrid sociolinguistic analytic method served as the basis for our analysis.^24^ Our analysis focused on verbal and non-verbal content although analysis of non-verbal aspects was limited in the audio-recorded PRP sessions. We used a transcription-free method of analysis, also known as ‘scribing’.^29^ The analytic process began with individual analysis by each team member of two randomly selected recordings (one BBN and one BMI). Thereafter, we met to discuss and reflect on our observations, and each team member was asked to report on salient aspects in the recordings they analysed.

Author 2 (J.B.) then analysed all 45 scenarios and the other authors each analysed a third of the dataset. Thus, each recording was analysed by two team members to promote trustworthiness. We conducted our analysis individually through repeated observation of the recordings, documented via reflective notes.

We began with a broad analytic approach addressing the following pre-developed questions for each recorded PRP scenario:

- What are the most salient features of this scenario?
- What’s working? That is, what communication skills used by the student in the role of doctor had a positive impact on the efficiency of the scenario and/or achievement of the communication task and/or relationship between the student-doctor and the patient role-player?
- What’s not working? That is, what communication skills used by the student in the role of doctor had a negative impact on the efficiency of the scenario and/or achievement of the communication task and/or relationship between student-doctor and patient?
- What psychosocial or rapport-related issues were salient?
- What content and information exchange-related issues were salient?
- What issues related to shared decision-making were salient?

The team agreed on preliminary overarching themes from the data, loosely using thematic analysis principles.^30^ This was carried out over a series of meetings. After a detailed analysis of all notes, the analysis was refined to produce a set of overarching themes, which were confirmed by consensus. The communication skills required for the peer-role-play task, according to the marking rubric, were mapped onto the themes identified in the data (Table 1).

**TABLE 1:** Themes and sub-themes identified in the data set in relation to the skills included in the feedback rubric.

Theme	Sub-theme	Rubric elements
1. Basic information giving and clinical correctness	1.1. Understanding medical information	*Verbal communication:* Clinically sound, incorporating ethics and/or law, development of a hypothesis *Advanced skills:* Relevance
	1.2. Conveying the information clearly	*Verbal communication:* Clear speech, medical jargon, systematic, using clichés, incorporating guidelines *Advanced skills:* Clarification, relevance, summary
	1.3. Getting to the point	*Verbal communication:* Systematic, truth-telling, using clichés, incorporating guidelines *Advanced skills:* Clarification, relevance
	1.4. Checking patient understanding of the information given	*Listening:* Active listening, respond to cues *Advanced skills:* Clarification, reflection – content *Non-verbal reading*: congruence, facial expression, mannerisms, body posture, eye contact
2. Approach to communication: doctor-centred versus patient-centred talk	2.1. Communicative style	*Verbal communication:* Rapport, shared decision-making, truth-telling, biopsychosocial approach *Questions*: Leading questions, double-barrel questions, probing questions, closed-ended questions, open questions *Advanced skills:* Reflection – feelings, empathy
	2.2. Listening to the patient	*Listening*: Active listening, respond to cues, respond appropriately, prompt appropriately, do not interrupt
	2.3. Plan of action or resolution	*Verbal communication:* Shared decision-making, management plan *Advanced skills:* Confrontation, dealing with resistance
3. Providing psychosocial support and responding with empathy	3.1. Awareness of lifeworld and contextual issues	*Verbal communication:* Biopsychosocial approach, culturally sensitive, shared decision-making *Advanced skills:* Reflection – behaviour, empathy
	3.2. Responding with empathy	*Advanced skills:* Reflection – feelings, empathy, use of silence *Non-verbal communication (projecting and reading)*: Congruence, facial expression, mannerisms, body posture, eye contact
	3.3. Integrating information giving and emotional aspects	*Verbal communication:* Biopsychosocial approach *Advanced skills:* Reflection – behaviour, reflection – feelings *Breaking bad news*: Advance preparation, build a therapeutic relationship, communicate well, deal with patient and family reactions, encourage and validate emotions

### Trustworthiness

Utilising Shenton’s recommendations,^31^ credibility was obtained by combining a variety of analytical perspectives across the research team and prolonged observation of the data set. Peer debriefs among team members were also used. A description of the setting and a detailed explanation of the analytic procedures helped to promote transferability. Dependability was obtained by documenting the study procedure via meeting notes and in a research journal. An audit trail, thorough review and consolidation of the team’s thematic analysis via multiple reflexive debrief sessions were used to establish confirmability.

### Ethical considerations

Ethical clearance to conduct this study was obtained from the University of the Witwatersrand, Human Research Ethics Committee (Medical) (Ref: R14/49) (No: M210517). Permission was obtained from the Registrar of the University and the integrated primary care committee in the Department of Family Medicine. J.W., who did not know the students, verbally explained the study in person and invited students to provide consent for analysis of their recorded scenarios. A departmental administrator emailed a participant information sheet, and 24 participants provided written consent.

## Results

Students used scenarios for the BBN assignment that related to cancer (cervical, prostate, lung, brain), miscarriage, human immunodeficiency virus (HIV) diagnosis, infertility, diabetes, epilepsy, teenage pregnancy, kidney failure and ectopic pregnancy. Students’ scenarios for the BMI assignment related to diabetes, hypertension, smoking and drug and alcohol addictions.

We observed similar patterns and trends in students’ communication skills across the two data sets for the BBN and BMI PRP scenarios. We identified three overarching themes in the data related to our observations of students’ communication skills in the scenarios, namely: (1) Basic information giving and clinical correctness, (2) approach to communication: doctor-centred versus patient-centred talk and (3) providing psychosocial support and showing empathy. The major themes and sub-themes identified in the data are included in Table 1, together with relevant elements from the rubric used to provide feedback to students. (Note that S = Scenario.)

### Theme 1: Basic information giving and clinical correctness

In general, we observed that students struggled with basic information giving and exchange. Their difficulties related to understanding the medical content of the information they were trying to convey, conveying the information clearly, getting to the point when providing information and checking patient understanding of the information provided.

#### Sub-theme 1.1: Understanding medical information

We noted several instances where students struggled with the accuracy of the medical content of the information they were trying to convey. For example, one student incorrectly indicated to a patient, ‘You can’t drive ever again with epilepsy’ (S9 BBN), and another stated, ‘Type 2 diabetes means you’re born with a deficiency’ (S5 BBN). In a scenario involving a patient with stage four cancer, the student offered contradictory information, suggesting they did not understand the condition: they talked about the need for counselling since this is a terminal illness, but when the patient asked if they were going to die, the student responded saying they did not know and needed to conduct more tests (S6 BBN).

Sometimes these challenges with medical content seemed to relate to the complexity of the condition the students had selected for the PRP scenario – and in some cases, students unwittingly selected what turned out to be a more complex scenario than they could handle. For example, in a scenario that involved a patient with kidney failure, the discussion around the management of the condition moved to the need for a kidney transplant, but the student was unable to answer the patient’s questions. The student ended up promising the patient a kidney and then backtracked, leaving the plan of action unresolved (S17 BBN).

#### Sub-theme 1.2: Conveying the information clearly

Students struggled with explaining difficult concepts in simple language, offering explanations that were either filled with jargon or else too simplistic. For example, one student described a biopsy as ‘I took a piece of meat from your cervix’ (S6 BBN). In another example, a student asked if the patient was ‘willing to do an MVA [*manual vacuum aspiration for termination of pregnancy*]’ (S14 BBN) without explaining what the acronym meant and the patient had to ask what the student was referring to. The same student then used the term ‘hysterectomy’, and again, the patient had to ask what this term meant.

#### Sub-theme 1.3: Getting to the point

Moving between the bigger picture and the smaller details when giving information proved a particular challenge. Some students focused too strongly on the big picture, while others inserted unnecessary minor details into their explanations. For example, in a scenario involving the diagnosis of diabetes, a student subsequently began instructing a patient about adherence strategies such as ‘Put your tablets next to your keys to help you remember to take them’ (S5 BBN), which did not seem relevant to the task of giving the results nor to the patient’s concerns. Sifting through what is most important to convey, particularly when BBN, also seemed difficult. Some students gave too much information about the condition or introduced drastic treatment options ‘We do a surgery and just take out the womb and both the ovaries’ (S20 BBN) in the case of cervical cancer. In contrast, others gave very little information ‘I’m not discussing this further’ (S1 BBN) or instructed patients to do their own research.

Some students were able to establish the reason for the consultation early on, but in other scenarios, this was not clear. Occasionally, students would talk circuitously for several minutes before the patient’s condition was even mentioned. During BBN scenarios, we observed that some students took a long time to get to the diagnosis, delaying this task with small talk or irrelevant questions. Alternatively, they jumped into sharing results too quickly, and the information was given bluntly – for example, with statements like ‘Your baby’s dead’ (S19 BBN) right at the start of a scenario related to a miscarriage.

#### Sub-theme 1.4: Checking patient understanding of the information given

Some students did well with checking patient understanding, using strategies such as asking directly about patients’ understanding of conditions – for example, ‘What do you know about diabetes?’ (S5 BBN) or ‘Do you know what causes seizures?’ (S9 BBN). Other students did not check their understanding at all. In one example, the student did not explain to the patient what the diagnosis meant, even though the patient did not seem to understand the diagnosis. The patient asked ‘So I’m going to die?’ but the student ignored this question and instead answered ‘There’s a lot you can do’ and ‘There’s a good team here’ (S6 BBN).

We also noted that the students’ timing for checking understanding was occasionally inappropriate. For example, when BBN, patients were asked what they knew about a particular condition before the diagnosis had been mentioned.

Students used circuitous approaches to gauge patients’ knowledge of a condition, which was not particularly efficient. Some students asked questions which did not seem directly relevant – for example, in a behaviour change scenario about alcoholism, a patient was asked:

‘Have you picked up anything on uh drinking and driving, like accidents? Car accidents? How most people die due to, like are involved in accidents due to drinking and driving?’ (S6 BMI)

### Theme 2: Approach to communication: doctor-centred versus patient-centred talk

The students’ communicative style generally embraced a doctor-centred rather than a patient-centred approach. They found it difficult to truly listen to a patient and seek to understand their concerns, resulting in the absence of a clear resolution or plan of action and limited evidence of shared decision-making taking place.

#### Sub-theme 2.1: Communicative style

We noted a tendency towards a paternalistic, didactic communicative style, typified by the student dominating the consultation. At times, students’ communication became rather condescending – for example: ‘You understand, né?’ (S6 BBN) or ‘Remember you came here last week?’ (S6 BBN) with a reintroduction of themselves. Students tended to default to scripted approaches to communicative tasks rather than tailoring their communication towards what the patient was telling them, probably because this approach felt familiar.

Some scenarios seemed rather interrogative, particularly the BMI ones, where students tended to use sequences of questions with patients. In some instances, patients were asked multiple questions in succession without pauses for them to respond – for example: ‘How do you feel about your diet? Did you change anything? What did you eat in the last few days?’ (S18 BMI).

A few students were able to achieve a more open, collaborative, patient-centred communicative style. In these instances, students invited patients to ask questions, adopted body language that suggested a more inviting approach and (as will be discussed in sections below) sought to understand the patient’s perspective.

Occasionally, students displayed behaviours that showed they were able to reflect in the moment on their communication skills. For example, in one scenario where a student used a more didactic style and provided a lot of information to a patient, the student stopped and said, ‘No, I must give you time to process the news’ (S13 BBN), demonstrating insight into her communication style and the need to implement specific strategies that had been taught in lectures.

#### Sub-theme 2.2: Listening to the patient

Listening to patients and understanding their perspectives and concerns seemed challenging to students. For example, in some instances, although patients were invited to ask questions, the student did not respond to the patient’s questions or else gave a response that did not answer the question. Sometimes students offered statements rather than asking questions. Other times, students discussed a patient’s condition without finding out first what the patient understood or how they felt about it. We also observed instances where students discussed management options without seeking the patient’s input and sometimes without having obtained sufficient case history information.

#### Sub-theme 2.3: Plan of action or resolution

Some scenarios included evidence of a clear resolution, with a commitment on the part of the patient to change or try something (in the BMI scenarios) and/or a discussion of the next steps and treatment options (in the BBN scenarios). In these instances, students adopted a patient-centred collaborative approach typified by questions such as ‘What have you tried?’, ‘What do you think might work?’ or ‘What concerns do you have?’, followed by a discussion of practical support strategies.

Other scenarios either included some attempt by the student to offer a plan of action, albeit in a clumsy, illogical way or they simply ended with no resolution. In the BMI scenarios, students often neglected to discuss aspects such as relapse, withdrawal, a timeline for action or how long a patient might try out a recommendation.

In general, students struggled to negotiate a plan of action using principles of shared decision-making. This seemed to relate to their difficulties with understanding the medical information and their adoption of a doctor-centred approach with no negotiation or reflection about the implications of the recommendation for the patient. For example, patients were sometimes simply informed about a referral with no further discussion to solicit the patient’s view on the referral.

### Theme 3: Providing psychosocial support and responding with empathy

While some students were able to engage well in attempting to understand the point of view of the patient and offered relevant and appropriate empathic responses and psychosocial support, we observed that many students found these skills challenging. In general, students were unable to successfully integrate information giving and emotional aspects in the same scenario.

#### Sub-theme 3.1: Awareness of lifeworld and contextual issues

Some students did well with asking about contextually related aspects and lifeworld issues that are related to the patient’s condition. For example, they asked about family members and significant others who could offer support to the patient. Other students found it difficult to ask about lifeworld and contextual issues or the impact of a condition on a patient’s life. Some used vague questions to probe psychosocial issues – for example: ‘So how does this affect your life?’ (S17 BBN).

Some students showed insensitivity to cultural differences or offered inappropriate solutions to patients’ concerns. For example, in an infertility case, an African patient was told they should pursue adoption or find a sperm donor, both of which may not be acceptable solutions in this instance.^32^ In another example, the suggested solution for the impact of epilepsy on a patient’s employment was to apply for a disability grant, which is inappropriate as the grant amount would fall significantly short of her current salary.

#### Sub-theme 3.2: Responding with empathy

Some students were able to utilise empathic responses with confidence. For example, in two different scenarios where patients experienced a pregnancy loss, students reassured them that ‘this is not your fault’ (S18 BBN) and ‘you are not to blame’ (S19 BBN). In another scenario where a patient had a diabetes diagnosis, the student stated that it is a ‘very manageable disease’, and it is ‘good that we caught it early’ (S5 BBN).

In general, however, students seemed to struggle with emotional aspects and responding empathically when a patient raised a concern. In some scenarios, this was carried out albeit inconsistently. In other instances, a patient’s concern was responded to inappropriately. Returning to the example of the patient with epilepsy, when she raised a concern – ‘I’m worried about not driving again’ – the student’s response was ‘Let’s get you a grant’ (S9 BBN). Here, a practical solution was offered rather than an empathic response, which would have been more appropriate.

Students also seemed to struggle, particularly when patients resisted recommendations or a plan of action. In one instance, after a patient resisted intervention for uncontrolled diabetes, the student capitulated, saying, ‘It’s okay, we don’t have to move mountains today’ (S23 BMI).

#### Sub-theme 3.3: Integrating information giving and emotional aspects

In general, we observed that students struggled to integrate the information-giving aspects and the psychosocial and emotional aspects of the scenarios, instead tending to compartmentalise these skills. Scenarios in which a student was successfully able to integrate both aspects into a consultation were rare. This trend did not seem dependent on the type of conditions selected.

For example, students demonstrated difficulties with finding a suitable approach to discussing a plan of action, especially in the behaviour change scenarios. Some students were overly blunt and used scare tactics rather than motivational approaches, focusing too strongly on information giving and not enough on providing emotional support. In one scenario involving a patient with uncontrolled diabetes, the student told the patient ‘The more you leave your sugar to be high, uh, it can even lead to blindness’ (S13 BMI). Other students were too tentative or circuitous, focusing too strongly on the emotional aspects and not providing enough information to the patient. In other instances, patients were informed that they would be sent to a psychologist, without any exploration of how the patient was coping and whether this would be a suitable referral.

### Engagement with peer role-play scenarios

In addition to the themes identified in the data and described above, we observed that, in general, most students struggled to engage with the realism of the peer simulation scenarios. Students appeared nervous, and their scenarios felt overly rehearsed. They tended to focus too much on the process of the simulation task, resulting in communication that felt rigid and distant or acted, rather than genuinely open, engaging and empathic.

In some instances, the scenarios appeared scripted or over-rehearsed; in other instances, they felt under-rehearsed. Some students submitted audio-recorded scenarios in which it was clear that both the doctor and patient roles were read directly from a written script, rather than prepared and enacted. In one instance, also an audio recording, the patient’s manner of engagement with the scenario suggested they were a real patient who was living with the condition being discussed, rather than a student enacting the role of the patient as they had been instructed to do.

Only two students were observed to engage in a more genuine, realistic manner during their scenarios. These students worked together as a pair, and each took on the role of doctor and patient across both scenarios. In one instance, the student playing the patient began to cry during the scenario and showed genuine emotion. While it initially appeared that the students were able to engage well, closer scrutiny and analysis of the recordings revealed that in the role of the doctor, the students tended to focus too heavily on psychosocial and emotional aspects, with insufficient attention given to the informational aspects required.

## Discussion

Peer role-play holds potential as an experiential learning approach to promote the acquisition of communication skills for medical students. Our findings show that it offers opportunities for students to practice skills such as information giving and psychosocial support. However, concurring with findings in the literature, we suggest that PRP needs to be implemented carefully.

Students in our study demonstrated difficulties with basic skills such as conveying medical information to patients, often struggling with the accuracy, clarity and relevance of the information shared. Students tended to either overwhelm patients with too much detail or else provide insufficient information. Errors in medical content and unclear explanations highlight gaps in both foundational medical knowledge and the ability to communicate complex information effectively. These difficulties are compounded when students inadvertently select PRP scenarios involving conditions beyond their expertise, revealing a need for more structured guidance in choosing appropriate case complexities. These findings reflect the literature, which shows that medical students tend to prioritise clinical knowledge over communication strategies.^33^

Furthermore, students found it challenging to integrate medical information giving with aspects of counselling, thus adopting a communication style that appeared more doctor-centred than patient-centred. This is confirmed by Karnieli-Miller et al.’s^34^ analysis of student reflections on BBN encounters, which highlights that medical students may fail to address patients’ emotional and informational needs and that most students are not able to integrate skills related to providing information, dealing with emotions and discussing a treatment plan. Although previous literature suggests that PRP is useful for developing empathic student-patient responses,^9,19^ this is not what we found. We acknowledge, however, that the complexity of the scenarios chosen by the students, their limited understanding of some medical conditions, a lack of prescriptive practice scenarios and the familiarity students have with each other may have influenced our contrasting findings.

Encouragingly, the instances we observed where students demonstrated reflexive skills such as pausing mid-conversation to recalibrate or using ‘think aloud’ strategies suggest that students have the capacity for effective communication. However, to achieve this, they arguably require stronger scaffolding and more practice opportunities to implement these strategies consistently. Reflective debrief opportunities (e.g. using video playback techniques) could support students in recognising their doctor-centred tendencies.

Our findings show that using PRP for teaching and learning communication skills presents some challenges for students related to realism.^35^ Role-play is removed from reality, and students may struggle to engage with it in a realistic way, as evidenced in our study, where only a few students were able to take on the role of either doctor or patient in a convincing way, and the recorded scenarios tended towards over-rehearsal or underdeveloped performances. Students do need to engage and interact with simulated approaches with sufficient realism to learn from them.^36^ Alinier and Oriot^35^ argue that differences between simulation and the real world must be addressed with students to foster authenticity and achieve the intended learning objectives.

Our findings suggest that a greater developmental progression of teaching and particularly practising communication skills is needed. This teaching should adopt a formative approach that incorporates not only didactic lectures but also blended learning, interactive elements and observational experiences. The focus should be on tailoring interactions towards particular patient needs, rather than following a checklist-based approach. Our findings suggest that students require more opportunities to observe and practise these skills across diverse contexts.

Students would benefit from more extensive preparation on how to better engage with role-play exercises – for example, a drama therapist embedded in the department could prepare students more thoroughly for engagement in role-play exercises, be it with peers or standardised patients.^14^ Further, to enrich the use of role-play for training, a greater or more explicit focus on reflective practice via self-regulated learning principles is needed for all students.^37^

Not all of the students are English first-language speakers, which some may argue could have contributed to difficulties in expressing themselves clearly or conveying their intended meaning during the PRP scenarios. While this is an important consideration in the diverse context of South Africa, the nature of the graduate entry programme at our institution means that students have already acquired other degrees in English before entering the programme, and the language of instruction is English. Rather, our evidence suggests that the challenges experienced by the students in this study extend beyond merely a language issue. For example, the ways in which students responded emotionally to the ‘patients’, their level of cultural sensitivity and their ability to integrate psychosocial elements were often problematic.

Most importantly, our findings highlight the need for more time to be spent on communication skills in the curriculum, with continuous feedback on progress in developing these skills. Advanced skills such as counselling strategies need time to develop, with multiple opportunities for practice and careful reflection during the learning process. The transition from theory to practice in this regard needs to be carefully nurtured in an explicit, considered and scaffolded approach to teaching.

### Strengths and limitations

A limitation was that the data sets we analysed included a relatively small sample of students who consented to participate, compared to the total number of students in the class. This low response rate is partially explained by general research fatigue patterns around the same period.^38^ The students had the opportunity to improve on their recordings after the formative feedback; however, these recordings were not included in our analysis and could have changed the results. We acknowledge that those students who provided consent may be more aware of their communication skills than others in the class and may have been more inclined to participate in the study. We did not interview the students about their experiences of engaging with PRP, which would have added a useful dimension of insight to this study.

This study’s strengths lie in its detailed observations of recorded PRP scenarios, including specific examples of student performance, which offer rich qualitative data. These insights go beyond a surface-level or checklist approach to reveal nuanced aspects of students’ communication strengths and weaknesses and highlight opportunities for improving approaches to teaching these skills.

## Conclusion

Breaking bad news and BMI are two particularly challenging communication tasks that medical students must learn. Our findings show that making the transition from communication theory to practice may be difficult for students to achieve, and learning how to integrate these complex communication skills is not straightforward. It is therefore important to implement earlier training of medical students and offer more scaffolded opportunities for practice if we want to develop basic competency in communication and counselling skills.

It is also crucial to consider the role of Family Physicians in fostering students’ communication skills. Family Physicians, who are often involved in training undergraduate students and providing hands-on experience in district settings, must be aware of the challenges associated with tasks like BBN and BMI communication. Their awareness of these complexities can enable them to better support students during training, offering guidance, feedback and opportunities for practical application of communication skills. By integrating these communication skill-building aspects into their teaching and supervision, Family Physicians can play a key role in shaping future practitioners who are not only technically skilled but also competent in effective, empathetic communication with patients.

Future research could explore whether providing students with a structured scenario yields different findings. Additionally, it would be valuable to examine the development of students’ communication and counselling skills over an extended period, tracking their progress and incorporating their reflections on their experiences. Greater emphasis should also be placed on analysing students’ engagement with PRP in the role of the patient.
